# A transdisciplinary model to inform randomized clinical trial methods for electronic cigarette evaluation

**DOI:** 10.1186/s12889-016-2792-8

**Published:** 2016-03-03

**Authors:** Alexa A. Lopez, Caroline O. Cobb, Jessica M. Yingst, Susan Veldheer, Shari Hrabovsky, Miao-Shan Yen, Jonathan Foulds, Thomas Eissenberg

**Affiliations:** Center for the Study of Tobacco Products, Virginia Commonwealth University, 1112 East Clay Street, Suite B-08, Box 980205, Richmond, VA 23298 USA; Department of Psychology, Virginia Commonwealth University, Richmond, VA USA; Department of Biostatistics, Virginia Commonwealth University, Richmond, VA USA; Tobacco Center of Regulatory Science, Department of Public Health Sciences, Pennsylvania State University College of Medicine, Hershey, PA USA; Department of Public Health Sciences, Pennsylvania State University College of Medicine, Hershey, PA USA

**Keywords:** Analytical methods, Electronic cigarette, RCT, NNAL

## Abstract

**Background:**

This study is a systematic evaluation of a novel tobacco product, electronic cigarettes (ECIGs) using a two-site, four-arm, 6-month, parallel-group randomized controlled trial (RCT) with a follow-up to 9 months. Virginia Commonwealth University is the primary site and Penn State University is the secondary site. This RCT design is important because it is informed by analytical work, clinical laboratory results, and qualitative/quantitative findings regarding the specific ECIG products used.

**Methods:**

Participants (*N* = 520) will be randomized across sites and must be healthy smokers of >9 cigarettes for at least one year, who have not had a quit attempt in the prior month, are not planning to quit in the next 6 months, and are interested in reducing cigarette intake. Participants will be randomized into one of four 24-week conditions: a cigarette substitute that does not produce an inhalable aerosol; or one of three ECIG conditions that differ by nicotine concentration 0, 8, or 36 mg/ml. Blocked randomization will be accomplished with a 1:1:1:1 ratio of condition assignments at each site. Specific aims are to: characterize ECIG influence on toxicants, biomarkers, health indicators, and disease risk; determine tobacco abstinence symptom and adverse event profile associated with real-world ECIG use; and examine the influence of ECIG use on conventional tobacco product use. Liquid nicotine concentration-related differences on these study outcomes are predicted. Participants and research staff in contact with participants will be blinded to the nicotine concentration in the ECIG conditions.

**Discussion:**

Results from this study will inform knowledge concerning ECIG use as well as demonstrate a model that may be applied to other novel tobacco products. The model of using prior empirical testing of ECIG devices should be considered in other RCT evaluations.

**Trial registration:**

TRN: NCT02342795, registered December 16, 2014.

## Background

This study is a systematic evaluation of a novel tobacco product, electronic cigarettes (ECIGs) that have become increasingly popular since their market entry [1, 2]. ECIGs use an electrically-powered heating element to aerosolize a liquid that usually (but not always) contains a combination of propylene glycol, vegetable glycerin, flavorants, and the psychomotor stimulant drug, nicotine. Despite their popularity, little robust evidence is available regarding ECIG safety or effectiveness for cigarette smoking cessation [3–5]. Instead, assertions are made that ECIGs likely will reduce tobacco toxicant exposure, cause few adverse events, and lessen the risk of tobacco-caused disease by reducing cigarette use [6, 7]. In fact, the data addressing how long-term ECIG use influences toxicant exposure, user health, and concurrent cigarette smoking are very limited [4, 8–12], and the issue is controversial [6, 13–15].

These abovementioned issues can be addressed empirically using randomized controlled trial (RCT) methods. Importantly, the two available RCTs of ECIGs, whose primary outcomes were related to cigarette smoking cessation, have used ECIG products that had unknown nicotine delivery profiles [16, 17] and, in at least one of the two cases, the product likely was ineffective at delivering nicotine to participants [16]. Considering the role of nicotine delivery in withdrawal suppression and product acceptability for cigarette smokers [18–21], a better RCT strategy might be to include ECIG products that are known to approximate the nicotine delivery profile of a tobacco cigarette.

In addition to ECIG liquid nicotine concentration, other product characteristics (e.g., propylene glycol/vegetable glycerin ratio, device battery, device voltage) may influence ECIG effects on toxicant exposure, user health, and concurrent tobacco use [22–27]. Previous qualitative and survey-based studies reinforce the conclusion that there are meaningful differences in the effects of different ECIGs [28, 29], and these studies also demonstrate the need for research on product characteristics, acceptability, and nicotine delivery prior to selection of an appropriate ECIG model(s) for use in an RCT. Interestingly, some ECIGs are effective in reducing the desire to smoke despite delivering zero or negligible nicotine [30–32], while others are capable of delivering cigarette-like nicotine levels as well as reduction in tobacco/nicotine abstinence symptoms [21, 33, 34]. These data provide a rationale for assessing within a single RCT the role of ECIG liquid nicotine concentration while holding other product characteristics constant. In addition, to understand the effects of the placebo or zero nicotine delivery ECIG a non-ECIG control condition, such as a cigarette-like product that does not involving production and inhalation of an aerosol, is warranted.

Another important RCT design consideration is the population of study. While the notion that ECIGs are particularly effective as smoking cessation aids is challenged by previous RCT results [16, 17] as well as at least some longitudinal studies (e.g., [35]), there is growing evidence from a variety of sources that ECIGs can help smokers reduce their cigarette intake (e.g., [35–40]). For example, daily use of ECIGs among cigarette smokers is not associated with increased rates of cessation at a 1-year follow-up assessment but rather with significantly reduced smoking [35]. Importantly, the health effects of this ECIG-induced combustible cigarette reduction are uncertain. Taken together, these results provide a rationale for an RCT investigating the effects of ECIG use and the role of ECIG liquid nicotine concentration in combustible cigarette smoking individuals who are interested in reducing their combustible cigarette intake. These individuals would likely be motivated to use the study product throughout the evaluation period, thus enhancing the likelihood of detecting effects on cigarette reduction if indeed these effects exist. Therefore, the current protocol focuses on studying cigarette smoking individuals interested in reduction within a RCT-based design, rather than examining the role that ECIGs may have on complete cigarette cessation based on those less conclusive findings [16, 17, 35].

Additionally, appropriate safety assessment is crucial in a RCT-based design using a novel product like ECIGs. While many reporting requirements are specific to trials involving investigational drugs or devices [41], designs of this nature are crucial to comprehensively and frequently assess for adverse reactions and events, particularly those that may meet the definition of an unanticipated or serious problem. Maintenance and regular updating of an exhaustive list of expected effects associated with study participation is vital. This list may be informed by a comprehensive literature search that examines previous short-term clinical studies of ECIGs and other assessments of current ECIG side effects (e.g., qualitative examinations, [42, 43]; and/or data mining techniques, [44]).

Cognizant of these design concerns, the current RCT protocol to examine the effects of ECIGs among cigarette smokers (funded by P50DA036105) performed by the Virginia Commonwealth University Center for the Study of Tobacco Products (VCU CSTP) was developed. The study population includes daily cigarette smokers who are not current ECIG users and are not interested in smoking cessation but who are interested in reducing their combustible cigarette use. The ECIG device type, ECIG liquid used, and adverse events assessed were informed by an iterative and integrated research plan addressing each of the issues highlighted above. The ECIG device selected for the RCT was determined using results from analytical laboratory testing and modeling that systematically examined numerous parameters including ECIG battery voltage, cartomizer resistance, number of heating elements, and user behavior [20, 27]. The nicotine delivery profile of the selected device across several ECIG liquid nicotine concentrations was examined in clinical laboratory studies (see [33] for more detailed methodology). In these studies, ECIG-naïve cigarette smokers participated in a crossover design in which they completed two 10-puff bouts from an ECIG that contained either 0, 8, 18, or 36 mg/ml nicotine solution (70 % propylene glycol and 30 % vegetable glycerin). Under these laboratory conditions, there was a direct relationship between liquid and plasma nicotine concentration observed [33]. At the 36 mg/ml liquid nicotine concentration, the mean plasma nicotine increase observed immediately after the 10^th^ ECIG puff (12.5 ng/ml) [33] approached that observed after the 10^th^ tobacco cigarette puff (16 ng/ml) under near-identical laboratory conditions [32]. This result provides the rationale for 36 mg/ml as the higher concentration in this study and the lower concentration of 8 ng/ml; a placebo (0 mg/ml) condition is included to examine the importance of nicotine on study outcomes. To examine the role that inhaling any ECIG aerosol (nicotine-containing or not), this RCT includes a cigarette substitute that produces no aerosol and neither contains nor delivers nicotine (cigarette-like plastic tube with air flow control to help achieve a similar draw resistance to a cigarette). Results from a qualitative examination of ECIG users were used to inform the potential adverse effects to be assessed in the current RCT [43]. Concept mapping, an integrative mixed method participatory approach that incorporates group-level processes and multivariate analyses to identify latent constructs, was used to identify concepts related to potential adverse events. These items were added to the side effects list assessed at each in-person visit for the current RCT.

### Aim

The specific aims of the RCT are to:

1. *Characterize ECIG influence on toxicants*, *biomarkers, health indicators, and disease risk.* We will measure exposure to the carcinogenic nitrosamine 4-(methylnitrosamino)-1-(3-pyridyl)-1-butanone [NNK; via its metabolite NNAL (4-(methylnitrosamino)-1-(3-pyridyl)-1-butanol) in urine], expired air carbon monoxide (CO), and nicotine (via its metabolite cotinine in urine). We will also measure heart rate and blood pressure, biochemical and hematologic health indices, pulmonary function (via spirometry), and biomarkers of oxidative stress. With respect to toxicant exposure, we hypothesize that, relative to the cigarette substitute condition, we will observe ECIG liquid nicotine concentration-related decreases in urine NNAL and expired air CO concentration.
2. *Determine the tobacco abstinence symptom and adverse event profile associated with real-world ECIG use*. We will use standard measures of nicotine/tobacco abstinence symptoms to characterize the extent to which ECIG-induced suppression of abstinence symptoms is related to nicotine concentration. With respect to other adverse events, we will assess effects likely attributable to inhalation of propylene glycol and nicotine self-administration. We hypothesize more of these propylene glycol-related adverse events with ECIGs relative to the cigarette substitute condition.
3. *Examine the influence of ECIG use on conventional tobacco product use.* We will monitor ECIG and all other tobacco/nicotine product use closely, via daily tobacco use diaries and in-person assessments. Because we are targeting individuals interested in reducing their cigarette intake, we hypothesize that individuals randomized to higher ECIG nicotine concentrations (8 mg/ml or 36 mg/ml) will experience reductions in combustible cigarette use.

## Methods/Design

### Design

The study will be a two-site, four-arm, 6-month, parallel-group randomized controlled trial with a follow-up to 9 months. VCU is the primary site with Penn State University (PSU) College of Medicine serving as the secondary site.

### Study population

Tobacco cigarette smokers throughout the greater Richmond, Virginia (primary site) and Hershey, Pennsylvania (secondary site) who report that they are interested in reducing their cigarette consumption and are not planning to quit smoking in the next 6 months.

### Inclusion and exclusion criteria

For inclusion in the study, participants must be between the ages of 21–65. They must report smoking >9 regular filtered cigarettes or machine-rolled cigarettes with a filter for at least 1 year and present with an expired air CO measurement of >9 parts per million at baseline. Participants must have made no serious cigarette smoking quit attempt in the prior 1 month. This criterion includes the use with the intent to quit cigarette smoking of any FDA-approved smoking cessation medication (varenicline; bupropion used specifically as a quitting aid; nicotine patch, gum, lozenge, inhaler, or nasal spray) in the past 1 month. While participants must not be planning to quit smoking in the next 6 months, they must report that they are interested in reducing their cigarette consumption by at least half in the next 6 months. They must be willing to attend visits weekly and monthly over a 9-month period (i.e., not planning to move, take an extended vacation, undergo surgeries). Finally, they must be able to read and write in English and be able to understand and give informed consent.

Participants will be excluded from the study if they are pregnant and/or nursing. Additionally, any unstable or significant medical condition in the past 12 months will lead to study exclusion (e.g., recent heart attack or some other heart conditions, stroke, severe angina including high blood pressure if systolic >159 or diastolic >99 observed during screening). Other health indicators for exclusion include immune system disorders, respiratory diseases (e.g., exacerbations of asthma or COPD, require oxygen, require oral prednisone), kidney (e.g., dialysis) or liver diseases (e.g., cirrhosis), or any medical disorder/medication that may affect participant safety or biomarker data. Use of any non-cigarette nicotine delivery product (e.g., pipe, cigar, dip, chew, snus, hookah, ECIGs, strips, sticks) in the past 7 days is also exclusionary at the initial baseline assessment. Other exclusion criteria include uncontrolled mental illness or substance abuse or inpatient treatment for these in the past 6 months, history of difficulty providing or unwilling to provide blood samples (e.g., fainting, poor veins, anxiety), surgery requiring general anesthesia in the past 6 weeks, use of an ECIG for 5 or more days in the past 28 days, use of marijuana or other illegal drugs daily/almost daily, or weekly in the past 3 months, use of hand-rolled roll your own cigarettes, any known allergy to propylene glycol or vegetable glycerin, and another member of household participated or currently participating in the study.

### Recruitment

Interested individuals will identify themselves by responding to advertisements for cigarette smoking research studies. Participants will be recruited via websites, message boards, radio and print advertisements, and web-based advertisement (e.g., craigslist.org). Before scheduling an assessment visit, all interested individuals will complete a screening questionnaire over the phone. Once an individual is deemed potentially eligible from the phone screener, an in-person assessment will be scheduled. An identical version of the phone screener will be performed first at the in-person visit. Following eligibility confirmation from the in-person screener, an informed consent and initial assessment visit that includes additional eligibility criteria will be performed. The study will be explained fully to eligible potential participants by the researcher during the informed consent process, and after being given adequate time to read the information and ask questions they will be asked to sign and date the informed consent form.

### Randomization, allocation concealment, and sequence generation

The four conditions are a cigarette substitute that does not produce aerosol, 0 mg/ml nicotine content ECIG (placebo), 8 mg/ml nicotine content ECIG (low), and 36 mg/ml nicotine content ECIG (high). Blocked randomization is accomplished with a 1:1:1:1 ratio of condition assignments at each participating institution with a goal of 260 randomized per site (*N* = 260 for VCU and PSU; *N* = 520 total). The assignment codes are made from separate randomization lists created in advance by the statistician for each site stratum. The randomization lists are stored in a secure, password protected drive and are shared only with unblinded staff. Once a participant has been confirmed eligible for randomization, a computer procedure will assign the participant to the next condition on the list automatically.

### Blinding

Unblinded staff at each site will fill individual cartomizers with ECIG liquid appropriate to their condition/flavor preference and then package the cartomizers into packs/kits for the participants. The blinded research assistant will not be involved in the packaging and labelling of the kits. The blinded research assistant will be given kits containing cartomizers specific to each participant that are only labeled with the visit identifiers, the participant ID, randomization ID, and expiration details, which will maintain the blind. Randomization to the cigarette substitute will be unblinded as this product looks and operates differently compared with ECIGs.

### Study interventions

**Fig. 1:**
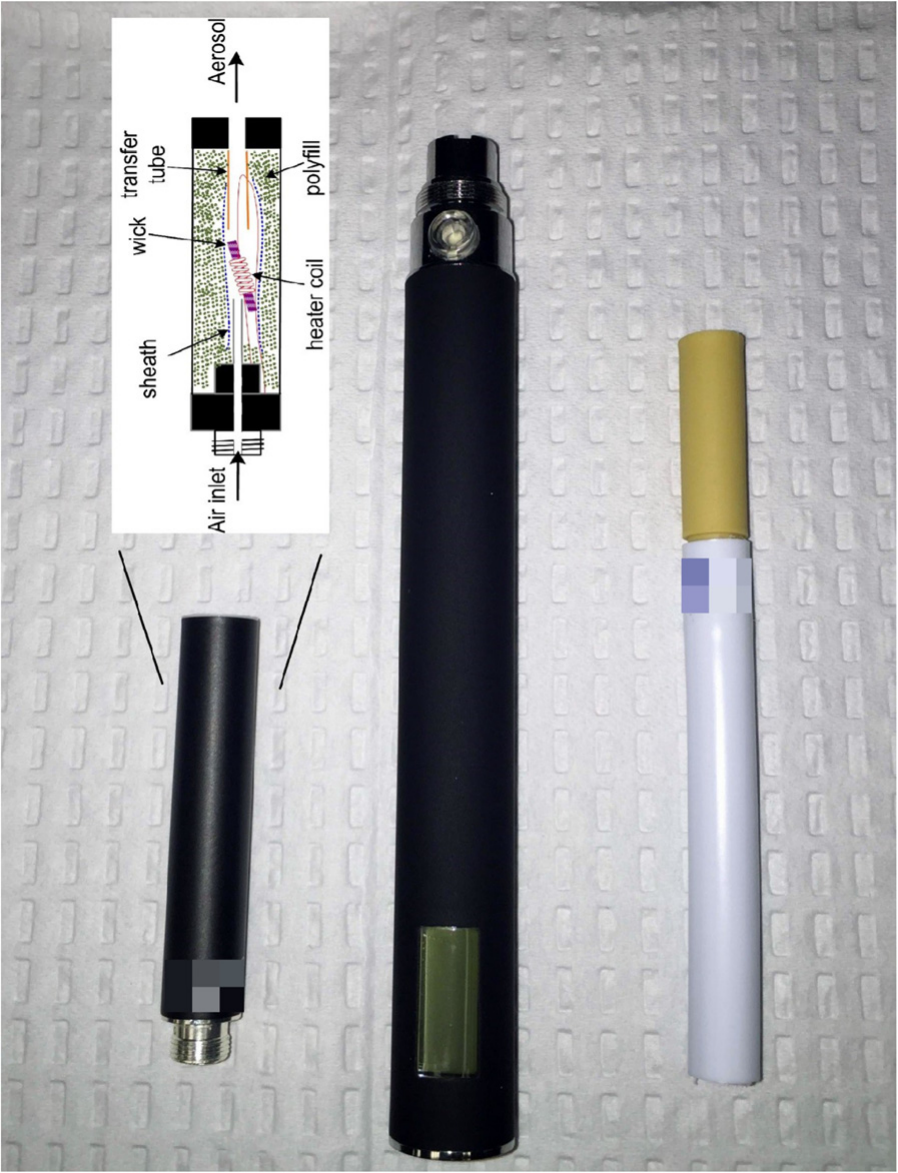
Study products with cartomizer schematic. The cartomizer, battery, and cigarette substitute are shown here (L to R). There is also a schematic of the cartomizer generously provided by Dr. Alan Shihadeh

Participants will be assigned to either the ECIG or the cigarette substitute (see Fig. 1). The ECIG consists of a 3.3 V, 1000 mAh battery (SmokTech; Shenzhen, China) with a 1.5 Ohm, dual-coil, 510-style cartomizer (SmokTech; Shenzhen, China) that is purchased without any liquid in it. Study staff load the cartomizer with 1 ml of a flavored (tobacco or menthol) 70 % propylene glycol/30 % vegetable glycerin liquid containing nicotine concentrations of 0, 8, or 36 mg/ml. The liquid is purchased from a local retailer (AVAIL; Richmond, Virginia, USA) who mixes it on site according to investigator specifications; the nicotine concentration is verified by an independent laboratory at VCU for each batch of liquid received (+/- 2 mg/ml). Each cartomizer is individually filled by staff to ensure the strength of the nicotine concentration, the amount of liquid placed into each cartomizer, and how recently it was filled. The cigarette substitute is a plastic, patented tube that looks like a real cigarette and is designed to provide the same draw resistance as a smoker’s usual cigarette (QuitSmart, Inc., North Carolina, USA). The airflow is controlled by a porous plastic insert located inside the cigarette tube and by a small ventilation hole between the posterior end of the cigarette filter and the anterior end of the cigarette tube. This device contains no nicotine or tobacco and produces no aerosol.

### Study visits

**Fig. 2:**
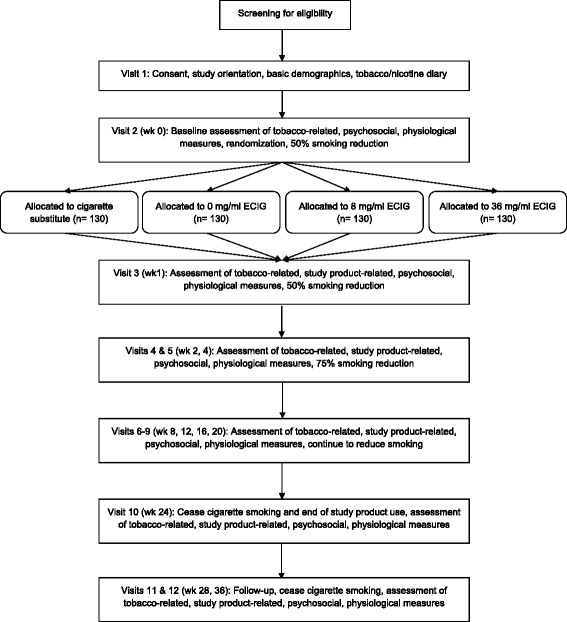
Study design including timeline for the four-arm, 6-month, parallel-group randomized control trial with a follow-up to 9 months

The sequence for all study visits, from screening to follow-up, are shown in Fig. 2. All participants who complete the phone screener are assigned a unique participant identifier (ID). Prior to informed consent procedures, potential participants will complete the screening questions that were completed previously over the phone to confirm no changes in eligibility. If a potential participant meets eligibility criteria they will continue with Visit 1 and IRB-approved informed consent will be obtained. After baseline assessments are completed, participants will return for Visit 2 following a week of recording of their regular cigarette smoking via a daily paper diary and no other intervention. This baseline period ensures that participants meet eligibility criteria (i.e., smoke >9 cigarettes per day), they are able to comply with study procedures prior to randomization (i.e., daily diary use, no use of any other tobacco product), and confirm that no quit attempts or use of smoking cessation medication have been reported. Participants unable to comply with these instructions or meet withdrawal criteria at the end of this first week will be excluded from further participation, and other individuals will be recruited to replace these participants who were withdrawn prior to randomization.

At the beginning of Visit 2, participants’ diary and a 7-Day Timeline Follow Back (TLFB; [45]) of cigarette smoking behavior will be examined by the research assistant to confirm the number of cigarettes smoked per day are >9 (for at least 5 out of the last 7 days). Following this procedure, eligible and able participants will be assigned to one of four experimental conditions following the block randomization scheme as described above for a period of 24 weeks using a unique randomization ID generated by a computer. Participants will receive the assigned study product (ECIG or cigarette substitute) and an associated user manual, and research staff will explain how to use the products. Each participant randomized to an ECIG condition will receive two batteries, one wall adapter, one USB charger, a user manual, and a carrying pouch. Participants will be allowed to test each ECIG liquid flavor briefly (tobacco and menthol) before selecting their ECIG liquid flavor to be used for the duration of the study. They will then be provided with cartomizers (approximately 3/day), pre-filled with liquid matched to their preferred flavor and concentration in childproof bottles. Participants randomized to the cigarette substitute condition will also receive two products, a user manual, and a carrying case. Supply of cartomizers and replacement of products will continue throughout the 24-week intervention period.

At Visit 2, randomized participants will complete tobacco-related and psychosocial study measures, and biomeasures will be collected. Tobacco-related measures include assessments of dependence, and psychosocial measures include the Kessler 6 [46], Perceived Stress Scale [47], and Center for Epidemiologic Studies-Depression scale [48]. Biomeasures include expired air CO, a pulmonary function test (PFT), exhaled breath condensate collection, and blood and urine samples. Blood samples will be submitted to the VCU/PSU hospital laboratory for a complete metabolic panel, hematology panel, lipid panel, and C-reactive protein assessment. An additional blood sample will be processed and stored for later analysis of oxidative stress markers. Urine samples will be stored for later analysis of cotinine, NNAL, and oxidative stress makers, and exhaled breath condensate will also be stored for later analysis for oxidative stress markers.

At the conclusion of Visit 2 participants will be instructed to start reducing cigarette smoking by 50 % starting the next day. The researcher will discuss with the participant ways to reduce cigarette consumption (e.g., only carrying the allowed number of cigarettes). Participants also will be instructed that they will be expected to complete daily diaries related to their study product and cigarette use. Visit 2 begins the intervention phase (study week 0) and the 50 % reduction phase of the study will continue through Visit 3 (study week 1) and Visit 4 (study week 2), with instructions for 75 % cigarette reduction given at the conclusion of Visit 4. At Visit 5 (study week 4), biomeasures will be completed again excluding hospital laboratory testing of blood sampled.

The continuation phase of the study is from Visit 6 (study week 8), Visit 7 (study week 12), Visit 8 (study week 16), Visit 9 (study week 20), through Visit 10 (study week 24). At each visit, participants will complete appropriate study measures and will be instructed to continue to reduce their cigarette consumption. At Visit 7 and Visit 10, biomeasures will be completed, but hospital laboratory testing of blood is only completed at Visit 10. At the conclusion of Visit 10, study product supplies will cease as well as use of the daily diary. Participants will be advised to cease all cigarette use and provided with freely available evidence-based smoking cessation resources. Those participants who were randomized to the cigarette substitute condition will be eligible to receive an ECIG with one week’s worth of cartomizers at 0 mg/ml; those randomized to an ECIG condition will be eligible to receive a cigarette substitute. This procedure, outlined during the consent process, is designed to increase the likelihood of retention for any participants who may have joined the study to reduce their cigarette consumption with an ECIG but were randomized to receive the cigarette substitute. Participants return during the follow-up phase at Visit 11 (study week 28) and Visit 12 (study week 36). Please note at each visit following consent, adverse events/medication changes and study product side effects (among other study measures) are assessed, and a 7-day TLFB is administered to assess cigarette and study product use. Participant compensation consists of $20 gift cards at each visit attended and an additional $20 gift card at visits where biomeasures are obtained; a bonus of $80 is given at the conclusion of the Visit 12 for those who complete all study visits.

### Primary outcome

The primary outcome measure of this RCT is the urinary concentration of the carcinogen biomarker of tobacco exposure, NNAL.

### Secondary outcomes

The secondary outcome measure will be urine cotinine concentration. Markers of oxidative stress (Glutithione and 8-Isoprostanes) are additional secondary outcome measures. Glutathione will be measured via blood sample analysis. 8-Isoprostanes will be measured via both urine sample and exhaled breath condensate analyses.

### Sample size

RCT power is based on the important biomarker of toxicant exposure, NNAL concentration in urine. Unfortunately, there are no data that reveal the NNK exposure in ECIG users, as that study has not yet been performed. However, we do have data showing how NNAL concentration in novel tobacco product users compares to own brand cigarette use [49]. Using these data, power analysis revealed 100 completers per condition would provide an 80 % power to detect an effect size of 0.28 pmol/ml (SD = 0.6 pmol/ml) on NNAL. We aim to enroll 130 participants per condition anticipating a 20 % attrition rate.

### Withdrawal criteria

There are numerous criteria in place for withdrawal consideration. Non-compliance with study procedures (e.g., consistently missing visits, not maintaining their daily dairies, unable to use their study product for a period of more than two weeks not by choice) are grounds for PI-consultation and withdrawal. Furthermore, participants will also be withdrawn from the study if they present with serious clinical adverse events, pregnancy, recurring illness, a new illness that could adversely interact with study participation, or worsening substance abuse. Any hospitalization at any time during participation is also grounds for study withdrawal. Hospitalizations will be self-reported by the participant and will be reviewed by the site PI and medical professional to determine whether continued participation in the study is appropriate (e.g., recovery from a major surgery, worsening of psychiatric symptoms, etc.). Finally, if a participant is behaving in an inappropriate or threatening manner, admits to dishonesty about eligibility criteria, is participating in other smoking research studies that could affect the primary outcome measures, appears/admits to giving away/selling study products, consistently loses study products etc., then they may be withdrawn from the study at the PI’s discretion.

Participants may also choose to remove themselves from the study by informing the research team in writing at any point during the study. If they choose to remove themselves from the study, they will not receive any further contact from the study site.

If participants are withdrawn from the study for any of the reasons noted above prior to randomization, they will be replaced until a total of 260 participants have been randomized to the study at each study site (VCU and PSU). Reasons for withdrawal will be ascertained from participants who withdraw from the study.

### Data management and monitoring

The protocol will not be initiated until it has received full approval by the relevant IRBs at each study site. All research staff that regularly come in contact with study participants will be instructed on procedures for managing and reporting adverse events. All adverse events will be documented, and records regarding the events will be retained for review by the study team. The VCU investigator will be informed immediately, in person or by telephone, of any potential or actual unanticipated problems at VCU, and they will report all unanticipated problems to the IRB at VCU within 5 days. VCU will also report unanticipated problems that occur at PSU to the VCU IRB within 5 days of becoming aware of the problem.

The study will be monitored by a Data and Safety Monitoring Board (DSMB) established at VCU and composed of four scientists not otherwise affiliated with the clinical trial. They will have expertise in substance abuse, psychiatric co-morbidities and biostatistics. The DSMB will meet (in person or via tele/video conference) once prior to the start of the study and at least semi-annually. Prior to the start of the study, the DSMB will establish a charter including study stopping rules and unblinding rules and procedures.

The Study Coordinator will generate a semi-annual report of aggregate data (including data collected at VCU and PSU) to be circulated to the PIs, project Co-investigators, and the four DSMB members. The report will summarize rates of recruitment, participants’ demographic characteristics, adverse events, unanticipated problems, serious adverse events (as defined the FDA), participant drop-out rates, cigarettes smoked per day, study product used per day, and expired air CO levels, and protocol deviations/violations. DSMB members will have one week to review the semi-annual report materials and either recommend that recruitment continue as planned or request a face-to-face meeting of the PI, the Study Coordinator, and all four DSMB members. If a DSMB meeting is convened, members will be asked to decide whether the study should: a) proceed as planned; b) proceed, but with a protocol amendment; or c) discontinue recruitment pending further investigation. If one or more DSMB members select option b or c, the PIs will inform their respective IRBs of such in a written report that is also copied to the FDA. Finally, a copy of the aggregate data report will be provided annually to the VCU and PSU IRBs.

Data security will be insured as follows. The majority of participant data will be collected using REDCap [50], a secure web application for collecting and storing research data hosted at VCU and made available through a collaboration between the VCU Center for Clinical and Translational Research and VCU Technology Services. To protect confidentiality, each participant will be assigned a participant ID number that will be used to identify all biological specimens. This unique participant ID number will also appear in the REDCap database associated with all data collected electronically.

### Statistical analysis

The analysis plan is based on the primary objective of determining the extent to which ECIG nicotine concentration influences NNK exposure as indexed by urinary NNAL concentration. We will first examine baseline characteristics including demographics and smoking history across study interventions to identify any baseline imbalances after randomization. Discrete variables will be summarized by frequencies and percentages and compared using Chi-squared test or Fisher’s exact test. Continuous covariates will be summarized by mean, standard deviation, median and range, and compared by one-way ANOVA. Skewed continuous variables will be log-transformed or square root transformed as appropriate. We expect groups to be balanced for important baseline characteristics due to randomization. A secondary analysis will be completed adjusting for age, gender, and race, along with any other covariates that differ across research interventions at baseline with a p-value less than 0.20. P-values less than 0.05 will be considered statistically significant with the exception of analysis of our primary analysis, where p-values less than 0.008 will be considered significant after a Bonferroni multiple comparisons adjustment. Our primary endpoint, urine NNAL concentration, will be summarized by study intervention and time and analyzed using linear regression, and we will adjust the Type I error rate to account for 6 pairwise comparisons at each time point. We will compare each ECIG group to the cigarette substitute and then each ECIG group to the other. An analogous approach will be used to analyze our secondary endpoints. The primary analysis of our secondary endpoints will use linear regression. Secondary analyses will consist of an adjusted analysis and a repeated measures analysis using a linear mixed model. All analysis will be completed using SAS (v.9.4) under the expertise of a senior biostatistician.

### Ethical considerations

Ethics approval was granted from the VCU and PSU Institutional Review Boards (VCU IRB: HM20002759; PSU IRB: STUDY00001095). Verbal consent will be obtained at the time of first contact over the telephone with the research team, and written consent will be obtained when participants attend their first study visit. All data will be entered, stored and backed-up in a secure manner via the REDCap database system.

## Discussion

This RCT uses a two-site, four-arm, 6-month, parallel-group design with a follow-up to 9 months. Participants are randomized to one of four conditions: a cigarette substitute negative control, or one of three ECIG conditions that differ by nicotine concentration 0, 8, or 36 mg/ml. The primary aims of this study are to characterize ECIG influence on toxicants, biomarkers, health indicators, and disease risk; determine tobacco abstinence symptom and adverse event profile associated with real-world ECIG use; and examine the influence of ECIG use on conventional tobacco product use. Liquid nicotine concentration-related differences on these study outcomes are predicted. These aims will address gaps identified by several reviews of ECIG-associated literature [3, 22, 51] and were chosen specifically for their relevance to proximal health effects as well as longer term health outcomes.

There were many factors considered during development of the current protocol. The two previous ECIG RCTs performed [16, 17] were important in that they demonstrated the challenges of ECIG product and control group selection. In response to this challenge, analytical and clinical laboratory testing were used to understand relevant ECIG device characteristics and nicotine delivery profile of the ECIG liquid nicotine concentrations chosen. The external analytic confirmation of nicotine content in all ECIG liquid used in the current RCT is also an important design characteristic and is in contrast to most clinical examinations of ECIG use [16, 17]. Prior knowledge of the liquid nicotine content and nicotine delivery capabilities of the device/liquid combination will strengthen the results obtained. Additionally, research staff at each site are responsible for filling individual cartomizers for this RCT. While more labor intensive, this procedural design detail allows for complete control and responsibility to ensure the integrity of liquid nicotine concentration in each participant’s cartomizers. As previously noted, we will test the liquid to confirm the nicotine concentration for additional safeguarding. We considered purchasing pre-loaded cartomizers, but decided the labor intensive approach was more appropriate given the importance of this element of the RCT.

In terms of control condition selection, the use of both an ECIG containing 0 mg/ml in addition to a control condition where no ECIG aerosol or nicotine is delivered (cigarette substitute) also is an important strength of the current design. Importantly, as this RCT is not a smoking cessation treatment study and we are not recruiting individuals interested in smoking cessation, the treatment-related equipoise considerations required of most RCTs are less applicable here. We see the cigarette substitute condition as a vital comparator to understand how ECIG aerosol and ECIG-delivered nicotine may affect user health among our other primary aims. While we anticipate the possibility that those assigned to the cigarette substitute condition could be more likely to withdraw their participation, Visit 10 procedures (ECIG provision to individuals who were randomized to the cigarette substitute) are intended to reduce this likelihood following the intervention period.

Another factor that needed to be considered when designing this protocol was the evaluation of side effects that ECIG users may experience. While the previous RCT trials highlighted potential adverse effect profiles of ECIG use, the RCT described here will also assess less well-known side effects related to ECIGs using results from recent qualitative work [43]. Learning from mixed design studies assessing potential adverse effects associated with ECIG use ensures that we are examining and characterizing both common and uncommon side effects related to ECIG use throughout the intervention and follow-up periods.

While there are a number of strengths regarding this RCT design, there have also been barriers to success that were faced during preliminary stages. In particular, there is not a universally agreed upon definition of an ECIG “use” within the literature and scientific community at this time. There are a number of mechanisms that can be used to measure cigarette and product use, such as daily text messages or the use of interactive voice response technologies. Daily diaries were chosen for this RCT due to the length of the intervention period and its complexity. In addition, the ECIG batteries included an automated puff counters on the ECIG which allowed for greater accuracy of use measurement. A related design challenge was the assessment of other tobacco product use and measures to assess ECIG use. There are few standardized measures of ECIG use [52], thus assessments for both during the intervention and follow-up phase needed to be developed and adapted from other sources. Future ECIG evaluations should consider these design aspects, specifically controlling the ECIG liquid used as well as the filling of cartomizers to ensure quality control.

Finally, this RCT design can inform the United States Food and Drug Administration’s (FDA) Center for Tobacco Products regulation standards for ECIGs and other tobacco products where cessation is not an indication (i.e., cessation is covered by FDA Center for Drug Evaluation and Research). The act giving the FDA its authority over tobacco products makes clear that regulation should be based on verifiable, scientific evidence. This science-based regulation involves evaluating the health effects of a potential tobacco product, especially the effects that might be expected under real-world use conditions. However, there are few demonstrated methods for predicting effects in the real world. This issue is becoming more critical as novel tobacco products are proliferating and evolving and major tobacco companies are revealing the pivotal role that novel tobacco products such as ECIGs play in their future business endeavors. ECIGs represent an important product category to examine and results from this study will inform knowledge concerning their use as well as demonstrate how this model may be applied to other tobacco products.

## Abbreviations

CO: Carbon monoxide
ECIG: Electronic cigarette
NNAL: 4-(methylnitrosamino)-1-(3-pyridyl)-1-butanol
NNK: 4-(methylnitrosamino)-1-(3-pyridyl)-1-butanone
TLFB: Timeline follow back
